# Investigation of concussion knowledge and attitudes of Chinese amateur adolescent soccer athletes

**DOI:** 10.1097/MD.0000000000033972

**Published:** 2023-06-09

**Authors:** Yue Li, Jiaxing Tang, Xiaomin Zhang, Dawei Cao, Teng Lyu

**Affiliations:** a School of Physical Education, Huaibei Normal University, Huaibei, China; b School of Physical Education, Shanghai University of Sport, Shanghai, China; c School of Electronic and Information Engineering, Huaibei Institute of Technology, Huaibei, China; d School of Physical Education, Huaibei Normal University, Huaibei, China; e Graduate School, University of Perpetual Help System Dalta, Manila, Philippines.

**Keywords:** adolescent soccer athletes, concussion, educational intervention, preventive strategies

## Abstract

Concussions are a common traumatic brain injury in soccer games but are often ignored by coaches and athletes. The purpose of our study is to assess the concussion knowledge and attitudes among amateur adolescent soccer athletes in China. Data was collected from questionnaire surveying (Rosenbaum Concussion Knowledge and Attitudes Survey (Student Version)) and semi-structured interviews completed by 69 amateur adolescent soccer athletes who participated in the U17 and U15 male groups of the 2022 China Youth Soccer League. The study followed a mixed methodology cross sectional study design. The concussion knowledge index (0–25) and concussion attitude index (15–75) scores were derived from the questionnaire and descriptive statistics were used for data analysis. The mean score of concussion knowledge is 16.8 ± 2.4 (range 10–22), and the mean score of concussion attitude is 61.3 ± 8.8 (range 45–77). Thematic analysis was used to categorize the participant’s responses of the semi-structured interview and the results were compared with their survey answers of questionnaire. Interestingly, the interviews revealed the inconsistencies between the questionnaire responses and intended behaviors, and multiple factors (injury severity, the importance of the game and substitution rules) influencing concussion-reporting behaviors were identified. In addition, athletes hope to acquire concussion knowledge through formal education. Our study lay the foundation for educational interventions to potentially improve concussion-reporting behaviors among amateur adolescent soccer athletes.

## 1. Introduction

Sports concussion is a brain injury caused by an external force.^[1]^ It accounts for about 5% to 9% of all sports injuries.^[2]^Soccer players are more likely to suffer concussions than other athletes due to the active use of the head during the game.^[2]^ A previous study indicated that approximately 35% of professional soccer players experienced at least a concussion and 23% experienced 2 to 5 concussions.^[3]^ A survey of professional football players in Canada revealed that 106 of the 454 respondents (23.4%) perceived they had suffered at least a concussion during their previous games or training.^[4]^ 82 concussions were reported in 194 Australian football matches.^[5]^ It is reported that there are annually over 50,000 concussions in high school soccer games in the United States.^[6]^ A concussion rate of 3.4 injuries per 10,000 athlete exposures was observed in high school women’s soccer, only second to high school football.^[7]^ Timely recognition of concussions is essential to prevent secondary impact syndrome. Second Impact Syndrome occurs when an athlete who has suffered an initial head injury, most often a concussion, sustains a second head injury before symptoms have fully cleared.” This second injury usually results in catastrophic brain swelling and fatal outcomes.^[8]^ Once an individual has suffered a concussion, there is a 3- to 6-fold increase in the risk of reoccurring concussions, which can lead to more severe complications and increased recovery time.^[9,10]^ In 2021, FIFA added extra substitutions to reduce the risk of concussions in soccer players.^[11]^ In the same year, the UEFA introduced a regulation (If a suspected concussion happens, the referee will call time out and allow the injured player to be evaluated by the team doctor) for the handling of concussions in matches.^[12]^ However, it is not uncommon for players to continue playing while sustaining concussions. For example, Pereira suffered a concussion after being hit at the 2014 World Cup but asked to continue to play despite the advice of the medical staff. He was frustrated when he was substituted. It is evident that the competitive situation may also increase the risk of concussion. A study demonstrate that more than 60% of concussions in Italian youth soccer go unreported.^[13]^ This may be because the player is unaware of the fact that he has suffered a concussion or does not believe that the injury is serious, or because the player does not want to withdraw from the game.^[14]^ These findings implicate there exists deficiencies of concussion knowledge and attitude among athletes, such as a lack of identification of concussion symptoms and risks associated with concussion complications.^[15]^ Currently found studies investigating concussion knowledge and attitudes mainly focus on football^[16]^ and baseball^[17]^ athletes. Moreover, to the best of our knowledge, there is no such studies conducted in China. Previous research has demonstrated differences between questionnaires and interviews.^[18]^ That was investigated the situation of sports activities in the past year through questionnaires and interviews, and the values of the questionnaire were significantly lower than those of the interviews. And the 2 methods to evaluate concussion knowledge and attitude were recommended.^[19]^ Educational interventions are used to influence people’s behavior through health education and popularization of safety awareness.^[20]^ Educational interventions can, on the one hand, increase the level of concussion knowledge among athletes and, on the other hand, reduce the rate of concussion underreporting by players.^[21]^ Interpretivist philosophy emphasize individuality and uniqueness, which induces the explanatory understanding of the personal experience and significance of the researcher in specific scenarios and event.^[22]^ The study is based on the interpretive theory and conduct a psychological measurement of Chinese amateur adolescent soccer players through a combination of questionnaire survey and semi-structured interviews, with aiming to assess their knowledge and attitudes about concussion and to provide evidence for educational intervention.

## 2. Methodology

### 2.1. Subjects of the survey

69 athletes (41 in the U17 category and 28 in the U15 category; age:15.9 years old ± 1.1 years; playing experience:8.4 years old ± 1.6 years) who participated in the U17, and U15 man group of the 1st China Youth Soccer League were selected for surveying by using a convenience sampling strategy. Consent was obtained from the coaches and parents, and all athletes provided written informed consent before writing the survey. The study was approved by the ethics committee of Huaibei Normal University. Two well-trained researchers, assisted by the medical staff of the organizing committee, given out the paper questionnaires and collected it immediately upon completion (recovery rate: 100%). Questions that were not understood by the athletes were explained by the research assistants without any leading comments. None of the researchers reported the existence of questions that could not be understood even after the explanation. No awards were given for this survey.

### 2.2. Research strategy

Questionnaires and semi-structured interviews were used to assess concussion knowledge and attitudes among amateur adolescent soccer players. The Concussion Knowledge Attitudes Questionnaire-Student Version (RoCKAS-ST)^[23]^was adopted; the semi-structured interview outline was developed based on previous studies^[23]^ and latest consensus statements of international conference on concussion in sport,^[1]^ and was screened by experts and reviewed by team physicians.^[24,25]^ To enhance the cross-linguistic equivalence of the instrument, the RoCKAS-ST questionnaire was translated into Chinese by using the classic inter-translation method. Two linguistic scholars proficient in English translated the English entries into the Chinese version (Step 1), and four proficient soccer professors corrected and revised the translated questions (Step 2). Then, two English major teachers who had not seen the original questionnaire translated it back into English (Step 3). The three steps were repeated until the semantics, expressions, and connotations of all the Chinese and English entries were matched. The RoCKAS-ST consists of 52 questions divided into five sections to test athletes’ concussion knowledge index (CKI, part I, II, and V) and concussion attitude index (CAI). The Part I of CKI includes 18 true/false questions (with one distractor item and three questions to monitor the validity of the questionnaire; with one point for a correct response and no score for an error; an invalid questionnaire was considered if the score is less than two); The Part II of CKI includes three situational judgment questions; The Part V includes 16 common concussion symptom identification options (with eight distractor items). The distractor items for common symptoms were replaced (e.g., hives, weight gain, difficulty speaking, over-learning, arthritis, hair loss, etc. were replaced with abnormal smell, abnormal taste, bruised eyes, neck pain, nosebleeds, etc.) since Saunders EA et al indicated that the replaced items were more reasonable.^[26]^ The total CKI score ranges from 0 to 25. The CAI consists of 15 questions rated on a five-level Likert scale (Part Ⅲ and Part Ⅳ). Score of 1 to 5 represents “strongly disagree-strongly agree” with a maximum score of 5 and a minimum score of 1. The total score ranges from 15 to 75. The RoCKAS-ST has been extensively tested for reliability and validity, and has demonstrated strong psychological applicability in practice.^[27]^

The semi-structured interview outline contains 27 questions. Players who had not received any concussion education before were pretested by the researchers. Participants were given parental and coaching consent and signed a written informed consent form prior to the survey. After completed the questionnaire, athletes respectively received anonymous semi-structured interviews.

### 2.3. Data analysis

The study adopted a cross-sectional design with a mixed methodology. The CKI (0–25) and CAI (15–75) scores were derived from the questionnaire and descriptive statistics were calculated with Microsoft Excel 2010 (Microsoft Corp., Redmond, WA). Interview transcripts were transcribed by the lead author and returned to the participants for confirmation. Irrelevant and duplicate data were removed, and responses from the semi-structured interviews were categorized and collated. The researchers conducted an inductive thematic analysis of the textual data collected from the semi-structured interviews. The text was transformed into coded statements for theme generation prior to analysis,^[28]^ following V. Braun and V. Clarke’s method of transcribing the interviews separately.^[29]^ First, the two researchers repeatedly read and discussed the interview text, and then coded the responses to each question item in groups, using Nvivol2 to assist with the coding. Second, the two researchers separately evaluated and named the core themes defined, with all responses considered. Then, the researchers discussed and determined the final naming of the themes. Finally, percentages were calculated based on the different themes and compared to the questionnaire.

## 3. Results

### 3.1. Attitudes to concussion knowledge (RoCKAS-ST)

All subjects completed the questionnaire and passed the validity screening (score 2.7 ± 0.5, inclusion criteria ≥ 2). Therefore, all 69 questionnaires were included for statistics and analysis. Scores on the CKI subscales are shown in Tables 1 and 2, with a mean score of 16.8 ± 2.4 (range 10-22). In both Part I and Part II of RoCKAS-ST (Table 1), the higher accuracy rate of common concussion knowledge included the following: concussions sometimes cause emotional confusion (94.2%, 65/69: S1 question 16); being diagnosed with a concussion presupposes that you have to be knocked down (89.9%, 62/69: S1 question 5); and symptoms of a concussion may last for several weeks (87%, 60/69: S1 question 8). The lower accuracy rate of common concussion knowledge included: symptoms usually disappear completely ten days after a concussion (20.3%, 14/69: S1 question 13); brain imaging (e.g., CAT scans, MRI, X-rays, etc.) after a concussion usually shows visible physical damage to the brain (e.g., bruising, blood clots) (21.7%, 15/69: S1 question 11); and Player Q’s concussion may affect his long-term health (34.8%, 24/69: S2 question 1). In part Ⅴ (Table 2), the mean score for correctly identifying symptoms was 5.7 ± 1.8 (range 0–8). The symptoms with the highest recognition rates were dizziness (92.8%, 64/69), headache (91.3%, 63/69), and amnesia (88.4%, 61/69). The symptoms with lower recognition rates were loss of consciousness (26.1%, 18/69) and sleep problems (50.7%, 35/69), with 53.6% of the subjects not selecting the distractor items.

**Table 1:** Concussion knowledge scores (CKI) (N = 69).

Questions	Correct (%)	Errors (%)
Part I		
1. If a second concussion occurs before the first has healed, there may be a risk of death.	**73.9 (51**)	26.1
3. People who have had one concussion are at greater risk of a second concussion.	**66.7 (46**)	33.3
5. To be diagnosed with a concussion you must be knocked down.	10.1	**89.9 (62**)
6. Concussions only occur when there is a direct impact to the head.	40.6	**59.4 (41**)
7. Being knocked unconscious usually causes permanent damage to the brain.	49.3	**50.7 (35**)
8. The symptoms of concussion may last for several weeks.	**87.0 (60**)	13
9. Sometimes, a second concussion can help a person remember things they forgot after the first one.	46.4	**53.6 (37**)
11. After a concussion has occurred, brain imaging (e.g. CAT scan, MRI, x-ray, etc.) will usually show visible physical damage to the brain (e.g. bruising, blood clots).	78.3	**21.7 (15**)
12. If you suffer a concussion for the first time, it will cause your intelligence to decline.	31.9	**68.1 (47**)
13. Ten days after suffering a concussion, the symptoms usually disappear completely.	**20.3 (14**)	79.7
14. After a concussion, people can forget who they are and not recognize other people, but are otherwise fine.	31.9	**68.1 (47**)
16. Concussions can sometimes lead to emotional turmoil.	**94.2 (65**)	5.8
17. An athlete suffering from a concussion can be knocked unconscious after a knockdown.	**84.1 (58**)	15.9
18. Multiple concussions rarely pose a risk to long-term health and comfort	29	**71.0 (49**)
Part II		
1. Player Q’s concussion could affect his long-term health.	65.2	**34.8 (24**)
2. Player X’s concussion could affect his long-term health.	**85.5 (59**)	14.5
3. Although Player F is still experiencing the effects of his concussion, he will perform as well as he would have as if he had not suffered a concussion.	34.8	**65.2 (45**)

This section shows the results of the responses to the 17 questions on concussion knowledge in the RoCKAS-ST; where bold font is the correct option and the number of correct answers is indicated in brackets; the total CKI score was calculated by adding the number of correct answers to these 17 questions to the number of actual concussion symptoms correctly identified.

CAT = computerized axial tomography, MRI = magnetic resonance imaging.

**Table 2:** Concussion symptom identification table (N = 69).

Symptoms	Current study (N = 69)%	A 2013 survey of US teens (Saunders et al, 2013a) (N = 150)%	A 2016 survey of British professional soccer players (Williams et al, 2016) (N = 26)%	A 2007 survey of US coaches (Valovich McLeod et al, 2007) (N = 156)%
Olfactory abnormalities	20.3	74.7	96.0	5.8
Taste abnormalities	21.7	75.3	100.0	7.1
Amnesia/memory loss	**88.4**	**64.7**	**52.0**	**60.3**
Blurred vision	**75.4**	**93.3**	**92.0**	**53.8**
Bruised eye socket	24.6	90.0	88.0	79.5
Chest pain	11.6	91.3	100.0	88.5
Confusion of consciousness	**73.9**	**94.0**	**92.0**	**89.1**
Dizziness	**92.8**	**94.7**	**92.0**	**88.5**
Headaches	**91.3**	**96.7**	**100.0**	**77.6**
Loss of consciousness	**26.1**	**90.7**	**80.0**	**80.1**
Nauseating	**73.9**	**71.3**	**64.0**	**55.8**
Nosebleeds	17.4	70.7	84.0	95.5
Numbness/tingling in upper limbs	13	49.3	92.0	82.7
Severe burning pain in the neck	14.5	64.0	96.0	89.7
Sleep disorders	**50.7**	**55.3**	**48.0**	**12.8**
Limited range of motion in the neck	27.5	57.3	56.0	10.9

Bolded font indicates actual concussion symptoms and the percentage (%) of subjects correctly identified; the current study is similar to the symptom options used in previous studies.

CAI subscale scores are shown in Table 3, with a mean score of 61.3 ± 8.8 (range 45–77). The safest concussion attitudes on the CAI subscale were: the athlete should have been taken to the emergency room after being knocked unconscious (84%, 58/69: S3 question 7); the coach needs to be very careful in deciding whether the athlete should return to play (75.3%, 52/69: S3 question 2); coach A made the right decision to exclude player R from the game (72.5%, 50/69: S4 question 1); Athlete H should have told the coach about the symptoms (72.5%, 50/69: S4 question 9); and most athletes agreed that Athlete H should have told the coach about the symptoms (72.5%, 50/69: S4 question 10). The riskiest attitudes toward concussion were: Most athletes agreed that the team doctor, not athlete R, should decide whether to allow a return to play (23.2%, 22/69: S4 Question 8); I feel that the decision to return to play should be made by the coach and not the athlete R. (15.9%, 11/69: S4 Question 7); I feel it is the athlete’s responsibility to return to play, even if it means still having concussion symptoms when playing (15.9%, 11/69: S4 Question 6).

**Table 3:** Concussion attitude score (CAI) (N = 69).

Questions	SD	D	N	A	SA
Part III					
1. When I have a headache due to a mild concussion, I will continue to exercise.	**46.4**	**24.6**	21.7	5.8	1.4
2. I think coaches need to be very careful when deciding whether an athlete should return to competition.	1.4	4.3	18.8	**33.3**	**42.0**
5. I don’t think concussions are as important as other injuries.	**26.1**	**43.5**	27.5	1.4	1.4
6. I feel it is the responsibility of the athlete to get back into the game, even if the symptoms of concussion are still present at the time of the game.	**33.3**	**26.1**	24.6	13.0	2.9
7. I think the athlete should have been taken to the emergency room after being knocked unconscious.	0	1.4	14.5	**33.3**	**50.7**
Part 4					
1. I think coach A made the right decision to leave player R out of the game.	2.9	2.9	21.7	**46.4**	**26.1**
2. Most athletes would feel that coach A made the right decision to exclude player R from the game.	1.4	2.9	29.0	**43.5**	**23.2**
3. I think Athlete M should make a comeback in the first game.	**18.8**	**40.6**	31.9	7.2	1.4
4. Most athletes would feel that Athlete M should make a comeback in the first game.	**21.7**	**40.6**	31.9	4.3	1.4
5. I think Athlete O should make a comeback in the semifinals.	**18.8**	**36.2**	39.1	4.3	1.4
6. Most athletes believe that Athlete O should make a comeback in the semifinals.	**23.2**	**31.9**	37.7	5.8	1.4
7. I think it should be up to the team doctor, not the athlete R, to decide whether or not to let the athlete R back into the game.	5.8	10.1	40.6	**30.4**	**13.0**
8. Most athletes agree that the team doctor, not the athlete R, should make the decision to return the athlete R to competition.	8.7	14.5	36.2	**26.1**	**14.5**
9. I think Athlete H should tell the coach about the symptoms.	1.4	0	26.1	**37.7**	**34.8**
10. Most athletes agree that Athlete H should tell his coach about his symptoms.	1.4	1.4	24.6	**37.7**	**34.8**

This section shows the results of the 15 Likert scale (1–5) responses on attitudes to concussion in the RoCKAS-ST; bolded font is the safer option, the safer the score the higher.

A = agree, D = disagree, N = neutral, SA = strongly agree, SD = strongly disagree.

### 3.2. Semi-structured interviews

Most of the athletes’ knowledge of concussions was derived from daily life (35%, 24/69) and partly from their own or teammates’ concussion experiences (28%, 19/69). 32% (22/69) of athletes had no knowledge of concussions. Most respondents perceived concussion as a blow to the head (62%, 45/69) accompanied by some common concussion symptoms. However, 17% (12/69) of the players reported incorrect concussion symptoms, such as brain hemorrhage, difficulty in moving, and nervousness. Almost all athletes felt that concussions were more serious than other sports injuries common in competitions (91%, 63/69) and that continuing to compete or train with a concussion was risky (93%, 64/69) and should be withdrawn from competition immediately (81%, 56/69). Nevertheless, this was contradicted by the fact that 62% of the athletes (43/69) chose to continue training and competing despite the possibility of concussion.

Most athletes felt that the importance of the game influenced their willingness to report a concussion (72%, 50/69), with almost all athletes choosing to hide the injury for fear of disappointing their teammates (93%, 64/69). There was disagreement in determining who should decide to “withdraw” or “return to play,” with 35% (24/69) of athletes saying it should be the coach’s decision, 25% (17/69) saying it should be their own decision, and 24% (17/69) saying it should be decisions of both sides. 12% (8/69) said it should be a joint decision among the team doctor, athlete and coach, and 4% (3/69) said it should be a team leader’s decision. There was also a divergence in opinions on how long an athlete should wait to play after suffering a concussion. Responses range from “one week” to “one month,” and most athletes said they should follow the advice of their doctor (75%, 52/69).

## 4. Discussion

Delayed treatment due to the untimely reporting of concussions has been a focus of concern in the sports medicine community.^[30]^ Catching the concussion knowledge and attitudes of athletes is important for improving concussion recognition and reducing the risk of concussion.^[31,32]^ Investigating athletes’ concussion knowledge and attitudes can provide targets for developing appropriate educational interventions. Questionnaires and semi-structured interviews indicated that amateur adolescent soccer athletes showed knowledge gaps of concussion and potentially risky behaviors. There are inconsistencies between the results if questionnaire and interview.

The CKI scores and CAI scores of the amateur adolescent soccer athletes investigated in this study were similar to that of British professional soccer players^[24]^ but lower than that of US college athletes.^[33]^ Common misconceptions of concussion knowledge include: the belief that imaging techniques can identify concussions; symptoms of concussions do not usually disappear completely after ten days; and concussions can affect long-term health. Some athletes who supported the above views in the semi-structured interviews believed that: “a brain scan in hospital would determine the extent of the injury”; “full recovery from a concussion could take six months or even a year”; and “the aftereffects of a concussion can affect future health.” Moreover, these athletes also felt that a concussion victim should be immediately taken out of play or training. It is interesting to note that those respondents who felt that recovery time from concussion was long and there were aftereffects indicated that concussion was a less serious injury than a “broken leg.” For the concussion symptom identification section, three symptoms were identified at a rate of more than 88%, with the lowest rates being “loss of consciousness” and “sleep disturbance.” This is consistent with previous studies.^[24,34]^ These results suggest that although there are many misconceptions about concussion knowledge, the majority of adolescent soccer athletes was able to identify common concussion symptoms. Surprisingly, athletes who are aware of the risks of concussion choose to ignore the symptoms.^[35]^

Amateur adolescent soccer athletes hold a positive attitude toward concussion. Most athletes felt that athletes should be taken to the emergency room after being knocked out, coaches need to be very cautious when deciding whether an athlete should return to play, and athletes should tell their coach about their symptoms in a timely manner. In contrast, only 38% of the interviewed athletes reported to stop training or playing if they experience a suspicious concussion. Therefore, using only a questionnaire to conduct a survey may lead to inaccuracies, as the options given in the questionnaire may limit players’ responses, and semi-structured interviews to some extent compensate for this limitation. These athletes in the interview stated, “I would try to keep playing and if I couldn’t, I would stop”; “I think I would recover quickly if I kept going”; “It’s up to the coach if I can keep playing.” This contrasts sharply with the consensus reached by the FIFA at the 4th International Concussion Conference, which requires that anyone suspected of having a concussion should be removed from the field of play immediately.^[36]^ However, none of the athletes interviewed indicated that they know these requirements. It is possible that there is a lack of access to concussion information for adolescent soccer athletes.

The extent that athletes take concussion seriously varies. Some believed that concussion is not serious. For example, athletes stated that “Concussion will not affect my performance in the game, because I am good at heading”; “concussions are not as serious as fractures”; and “concussions are faster to recover from than groin strains.” Others took it very seriously by stating that “Although my head is strong, my brain is the most important organ in the body”; and “A concussion sounds scary, and I will be very careful.” Yet they said later in the interview that they “would try to hang on and continue playing if they had a concussion during the game.” This is consistent with the findings of a previous study^[35]^ that athletes may conceal concussions during games. The majority of respondents believed that there were short-term or long-term risks associated with concussions. However, they were unable to articulate these risks other than “secondary damage” or “cognitive problems.” Therefore, concussion education is necessary to help athletes realize the dangers of concussion and to make them to report concussion symptoms in a timely manner.^[30,37–39]^Two extraneous factors that may influence concussion reporting are the “substitution rule” and the “importance of the game.”^[40]^ Almost all athletes said that the importance of the game influenced their decision to report a concussion, while 55.1% of athletes selected the option that “I would not return early because of semifinals” in the RoCKAS-ST questionnaire. Then, there is often a discrepancy between reported intentions and actual behavior. For example, in a game in which the concussion substitution rule is not enforced and in the situation that the team has exhausted its substitutions, the majority of respondents would remain in the game rather than put their team at a disadvantage due to that if the player came off the field at this point with a suspected concussion, the team would be playing with one less player than the opponent. In addition, unlike other sports, soccer matches do not allow much time for the medical team to diagnose the athlete’s injury. As a result, a quick evaluation is required to assess whether an athlete will continue to play or be replaced. This places tremendous pressure on the medical team. The Australian soccer league has a more humanistic protocol for concussion diagnosis, which requires a player with a suspected concussion to be observed for a minimum of 20 minutes. There is a 10-minute break, followed by a minimum 10-minute concussion assessment during which the replacement player can participate in the game. The Sport Concussion Assessment Tool was initially developed at the Second International Conference on Concussion in Sport in Prague in 2004 by combining existing tools such as the Glasgow coma scale (GCS), modified Maddocks Score, modified Post-Concussion Symptom Scale (PCSS), mechanism of injury and background information, Standardized Assessment of Concussion (SAC), and examinations of the neck, balance, and coordination.^[41]^ If the player passes the Sport Concussion Assessment Tool, referee will allow them to continue to play. If they do not pass the test, they will be substituted off the field,^[42]^ which would reduce the number of players who can play. This provision greatly reduces the risk that an athlete will face if they suffer a concussion. However, to date, this rule has not been introduced in Chinese soccer league at any level.

In competitive sports, playing with an injury’ and throwing oneself into the game immediately after an injury are considered tokens of courage and tenacity. Therefore, many players do not report a concussion even if they feel they may have one.^[43]^ Among them, some felt that the decision to come off the field should be based on their physical state. As they mentioned in the interviews, “If I have a concussion, but I’m still playing well, why should I be replaced?”; and “If I concentrate and keep playing and the symptoms go away, there is no need to come off the field.” Some athletes put the team interests above all else and said, “I’ll stick around for the team to win.” There are many cases of soccer players who have suffered concussions and continue to play.

Self-reporting of subjective and non-visible symptoms is critical to ensure proper concussion management. For this reason, educational interventions target focus on concussion reporting.^[38]^ Adolescent soccer athletes who have received concussion education are more likely to report the symptoms of concussion in the game.^[39]^ Educational interventions need to accurately assess the knowledge gaps in a particular population to develop relevant educational strategies.^[44]^ The knowledge gaps identified in our survey have important implications for the development of educational intervention strategies. As the soccer population in China continues to grow, there is an urgent need to develop concussion education programs for adolescent soccer players. Sports organizations and media should enhance the concussion education and publicity to improve knowledge and awareness of concussion among athletes. For example, sports clubs could hold concussion lectures and soccer stadiums set up bulletin boards for concussion knowledge. Our study found that athletes would play with a suspicious concussion, which is deleterious to athletes. Therefore, in addition to educational interventions on athletes, coaches or team medical staff should be literate in concussion identification. Meanwhile, management on concussed players should be strengthened, and a careful recovery plan should be formulated to avoid secondary injury.

## 5. Conclusion

Chinese amateur adolescent soccer players scored highly on concussion knowledge and attitude. However, semi-structured interviews revealed inconsistencies between the questionnaire responses and intended behaviors and potentially risky behaviors. This suggest that questionnaires may not be a valid tool for assessing concussion attitudes. Furthermore, athletes want to acquire concussion knowledge through formal education. Our study identified the interventional target for concussion education and provide helpful strategies to improve concussion knowledge and reporting behavior of adolescent athletes.

## 6. Limitations and prospects

Due to the time and staffing constraints of this survey, only a random selection of male U17 and U15 players with a single ethnic group from one division of the National Youth Soccer League were surveyed. In addition, concussion knowledge and attitudes of coaches and parents play and important role in the prevention, identification and management of concussion for adolescent athletes. However, this is not addressed in this study, which could be explored in depth in future studies.

## Acknowledgments

We thank all members of the research group and all athletes who participated in the survey for their support.

## Author contributions

**Conceptualization:** Jiaxing Tang.

**Formal analysis:** Yue Li.

**Funding acquisition:** Teng Lyu.

**Investigation:** Yue Li, Xiaomin Zhang, Dawei Cao.

**Methodology:** Yue Li, Jiaxing Tang.

**Project administration:** Yue Li, Dawei Cao, Teng Lyu.

**Software:** Xiaomin Zhang.

**Supervision:** Jiaxing Tang.

**Visualization:** Jiaxing Tang.

**Writing – original draft:** Yue Li.

**Writing – review & editing:** Teng Lyu.

## Supplementary Material


