# Supplementary material for: Investigation of concussion knowledge and attitudes of Chinese amateur adolescent soccer athletes
Source: Medicine (Baltimore). 2023 Jun 9;102(23):e33972. doi: 10.1097/MD.0000000000033972 (PMC10256388; doi:10.1097/MD.0000000000033972)
Supplement: Supplementary file 1 [file medi-102-e33972-s001.pdf]

number	source	age	Competition experience	@1如果在第一次脑震荡痊愈之前发生第二次脑震
1	U17	16	9	1
2	U17	16	8	2
3	U17	17	11	1
4	U17	16	10	1
5	U17	16	11	1
6	U15	15	7	1
7	U17	17	9	1
8	U17	15	11	1
9	U17	16	8	1
10	U17	16	11	1
11	U15	14	8	1
12	U17	17	8	1
13	U17	17	8	1
14	U15	15	6	1
15	U17	17	9	1
16	U17	17	10	1
17	U17	16	11	2
18	U17	17	8	1
19	U17	17	9	1
20	U17	17	10	2
21	U17	17	8	2
22	U17	17	10	2
23	U17	17	11	1
24	U17	17	9	2
25	U17	17	10	2
26	U17	17	10	1
27	U17	17	8	1
28	U17	16	10	1
29	U17	16	11	1
30	U17	16	9	1
31	U17	17	8	1
32	U17	17	10	1
33	U17	17	9	2
34	U17	17	11	1
35	U17	17	9	1
36	U17	17	11	2
37	U17	17	11	1
38	U17	17	10	1
39	U17	17	8	1
40	U15	15	8	2
41	U17	17	10	2
42	U17	17	8	2
43	U15	15	6	2
44	U15	15	7	1
45	U15	15	6	1
46	U15	15	7	1
47	U15	14	7	1
48	U15	14	6	1
49	U15	15	7	1
50	U15	15	8	2
51	U15	15	7	1
52	U15	15	7	2
53	U15	15	8	1
54	U15	14	7	2
55	U15	14	6	1

56	U15	15	6	1
57	U15	15	7	1
58	U15	15	7	1
59	U15	14	8	1
60	U15	15	8	1
61	U15	15	8	2
62	U15	15	6	1
63	U17	17	9	1
64	U17	16	7	1
65	U15	15	8	1
66	U15	15	6	1
67	U17	17	10	1
68	U15	15	6	2
69	U15	15	6	1

@2每天跑步对改善心血管健康作用不大。	@3有过一次脑震荡的人患脑震荡几率更大。
2	2
2	1
2	1
2	1
2	1
2	1
2	1
2	1
2	1
2	1
2	1
1	2
2	1
2	2
2	1
2	2
2	1
1	1
2	1
2	1
2	2
2	1
1	2
2	1
2	1
2	2
2	2
2	1
2	2
2	2
2	2
2	2
2	1
2	1
2	1
1	2
2	1
1	2
2	1
2	1
2	1
2	1
2	1
2	1
2	1
2	2
1	2
2	1
2	1
2	1
2	1
2	1
2	1
2	1
2	1

2	1
2	2
1	1
1	1
2	2
2	1
2	2
2	2
2	1
2	2
1	2
2	1
2	2
1	1

@4钉鞋能够给运动员提供更好的抓地力。	@5只有被击倒的情况下，才可能被确诊为脑震荡
1	1
1	2
1	2
1	2
1	2
1	2
1	2
1	2
1	2
1	2
1	2
1	1
1	2
1	2
1	2
1	2
1	2
1	2
1	2
1	2
1	2
1	2
1	2
1	2
1	2
1	2
1	2
1	2
1	2
1	2
1	2
1	2
1	2
1	2
1	2
1	2
1	2
1	2
1	2
1	2
1	2
1	2
1	2
1	2
1	2
1	2
1	1
1	2
1	1
1	2
1	2
1	2
1	2
1	2
1	2

1	2
1	2
1	1
1	1
1	2
1	2
1	2
1	2
1	2
1	2
1	2
1	2
2	2
1	1

@6只有头部受到直接撞击时，才会发生脑震荡。	@7被击昏通常会对大脑造成永久性损伤。
2	2
2	2
1	2
2	2
2	2
1	1
2	1
2	1
2	1
2	1
2	1
1	2
1	2
2	1
2	2
1	2
2	1
1	1
2	2
1	2
2	1
2	1
2	1
1	1
2	2
2	1
2	2
2	1
2	2
2	2
1	2
1	1
2	2
2	2
2	2
1	2
1	1
2	1
2	1
2	2
2	2
2	2
1	2
2	2
2	1
1	2
1	2
1	2
2	1
1	1
2	1
2	2
2	1
1	1
1	2

1	1
2	1
2	2
1	1
1	1
1	1
2	1
1	1
1	2
1	2
1	2
2	1
2	2
1	1

@8脑震荡的症状可能持续数周。	@9有时，第二次脑震荡可以帮助一个人想起第一
1	2
1	1
1	2
1	1
1	2
1	1
1	1
1	1
1	1
1	1
1	1
2	1
1	1
1	1
1	2
1	1
1	2
1	2
1	1
2	2
2	2
1	2
1	2
1	2
1	2
1	1
1	2
1	2
1	2
1	2
1	1
1	2
1	1
1	2
1	2
1	2
1	2
1	2
1	2
1	2
1	1
1	1
1	2
1	2
2	1
2	2
1	2
1	2
1	1
1	1
1	2
1	1
1	2
1	1
1	1

1	2
1	2
2	2
1	1
2	1
2	1
1	1
1	1
1	1
1	2
1	2
1	1
2	2
1	1

@10举重有助于强健或锻炼肌肉。	@11脑震荡发生后，脑成像（如CAT扫描、MRI、
1	1
1	2
1	1
1	1
1	1
1	2
2	2
1	1
1	1
1	1
1	1
1	1
1	1
2	1
1	1
1	2
1	1
1	1
1	1
1	1
1	2
1	1
1	2
1	1
1	2
1	1
1	1
1	1
1	2
1	2
1	1
1	1
2	1
1	1
1	2
1	1
1	1
1	1
1	1
1	2
1	1
1	1
1	1
1	1
1	1
2	1
2	1
1	1
1	1
1	1
1	1
1	2
1	1
1	1

1	1
1	2
2	2
1	1
2	1
2	1
1	1
1	1
1	1
1	1
1	1
1	1
2	2
1	1

@12如果你第一次患脑震荡，你的智力会下降。	@13十天后，脑震荡的症状通常会完全消失。
2	2
2	2
1	2
2	2
2	2
2	1
2	2
1	2
1	2
1	2
2	2
2	1
2	2
1	1
2	2
2	2
2	2
2	2
1	2
2	1
2	1
2	2
2	2
2	2
2	2
2	2
2	2
1	2
2	1
1	2
2	2
2	2
2	2
1	2
2	2
2	2
2	2
1	2
2	2
1	2
2	2
2	2
2	2
2	2
2	1
2	2
1	2
2	2
1	1
1	2
2	1
2	2
1	2
1	2
1	2

2	1
2	1
2	2
1	2
1	2
2	2
1	2
1	1
2	1
2	2
2	2
2	2
2	2
1	1

@14脑震荡后，患者可以忘记自己是谁，不记得认	@15高中新生和大学新生可能年龄相同。
1	1
1	1
2	2
1	1
2	1
2	1
2	2
1	1
1	1
1	1
2	2
2	1
1	2
1	2
2	2
2	2
2	1
1	2
2	2
2	1
1	2
2	1
2	1
2	2
1	2
2	1
1	2
1	2
2	1
2	2
2	2
2	1
2	2
2	2
2	1
2	1
2	1
2	1
2	1
1	2
2	1
2	1
2	2
2	1
2	2
2	2
2	2
2	1
2	2
1	1
2	2
2	1
2	2
1	1
2	2

1	1
2	2
2	2
1	1
1	2
2	2
2	2
1	1
2	2
2	2
2	2
1	1
2	2
1	1

@16脑震荡有时会导致情绪混乱。	@17运动员遭受脑震荡后出现神志不清，那他可能
1	1
1	1
1	1
1	2
1	2
1	2
1	1
1	1
1	1
1	1
1	1
1	1
1	1
1	1
1	2
1	1
1	1
1	1
1	1
1	2
1	1
1	1
1	1
1	1
1	1
1	1
1	1
1	1
1	1
1	2
1	2
1	1
2	1
1	1
1	1
1	1
1	1
1	1
1	1
1	1
1	1
1	1
1	1
1	1
1	1
1	1
1	1
1	1
1	1
1	1
1	1
1	1
1	1
1	1
1	1

1	1
1	1
2	2
1	1
1	1
1	1
1	1
1	1
1	2
1	1
2	2
1	1
2	2
1	1

@18多次脑震荡几乎不会对运动员以后的健康状况	@1球员Q的脑震荡可能会影响他未来的健康状况。
1	1
2	2
1	2
2	1
2	1
2	1
2	1
2	1
2	1
2	1
1	1
2	1
1	2
2	1
2	2
2	2
2	2
1	1
2	1
2	2
1	2
2	2
2	2
2	2
2	2
2	1
1	1
2	1
1	1
2	2
2	2
2	1
2	1
2	1
1	1
2	2
1	1
2	1
2	1
2	1
2	1
2	1
2	1
2	1
2	1
2	2
2	2
2	1
2	1
2	2
2	1
1	2
1	1
1	1
2	2

2	1
2	1
2	2
1	1
1	1
2	2
1	1
1	1
1	1
2	1
2	2
1	1
2	2
1	1

@2球员X的脑震荡可能会影响他未来的健康状况。	@3尽管球员F正受到脑震荡带来的影响，但他在比
1	1
1	2
1	1
1	1
1	2
1	2
1	2
1	2
1	2
1	2
1	1
2	1
1	2
1	1
1	2
1	2
1	2
1	2
1	1
2	2
1	1
1	2
2	2
1	2
1	2
1	2
1	1
1	2
1	2
1	2
1	2
1	2
1	1
1	2
1	2
2	2
1	1
1	2
1	1
1	2
1	2
1	2
1	2
1	2
1	1
1	1
1	2
1	2
1	2
1	2
1	2
2	1
1	1
1	1
1	2

1	1
1	2
2	2
1	1
2	2
2	2
1	1
1	1
1	1
1	2
2	2
1	1
2	2
1	1

@1虽然受到脑震荡后头疼的影响，但我仍然会坚	@2我觉得教练在决定运动员是否应该返回赛场时
5	5
4	4
5	5
2	4
4	2
4	4
4	4
5	4
5	4
5	4
3	3
3	3
4	5
4	4
5	5
5	5
5	5
5	5
2	5
5	4
4	4
3	5
5	5
5	4
4	5
5	5
3	4
5	5
5	4
5	4
5	5
5	5
5	4
3	4
4	4
5	5
2	3
5	5
3	5
4	5
5	5
5	5
4	5
3	3
4	4
5	5
5	5
5	4
5	5
3	5
4	4
5	4
4	3
3	3
5	5

3	4
3	3
3	3
3	3
5	4
5	5
5	3
1	3
4	1
3	3
4	2
2	5
4	2
3	3

@3我觉得护齿器可以保护牙齿不受伤害。	@4我觉得职业运动员比高中运动员更擅长他们所
5	5
4	4
4	4
4	4
5	4
4	4
4	3
4	4
4	4
4	4
5	3
3	3
5	5
5	5
5	5
5	5
4	5
5	5
4	5
4	4
5	4
4	3
5	3
3	5
5	5
3	4
4	5
5	5
5	4
4	5
5	5
5	4
4	4
4	5
5	5
3	5
4	5
5	3
5	5
5	5
5	1
5	2
4	4
5	5
3	4
5	5
5	3
4	4
5	3
4	4
4	4
4	4
4	4
3	3
5	4

4	5
4	3
3	3
3	3
4	3
5	4
3	3
1	5
5	5
4	5
2	5
5	5
2	2
3	3

@5我觉得患脑震荡不如患其他伤害严重。	@6我觉得运动员有责任返回赛场，即使后面的比
3	4
4	5
4	3
3	2
3	4
5	4
4	4
5	5
5	5
5	5
3	4
3	3
4	4
4	4
4	5
3	4
4	4
3	2
5	3
4	4
4	3
2	2
4	5
4	5
4	2
4	3
4	3
5	4
4	4
5	5
4	5
5	5
3	5
4	3
4	2
5	5
4	2
4	5
3	3
5	5
5	5
4	5
4	5
3	3
4	3
3	5
4	5
4	4
5	5
4	4
4	4
5	5
4	4
3	3
5	5

3	3
4	4
3	3
3	3
4	2
5	2
3	3
1	1
5	4
3	5
5	3
5	1
3	2
3	3

@7我觉得运动员受到撞击后神志不清，应该被立	@8我觉得大多数高中运动员将来都会成为职业运
5	4
4	2
4	3
5	3
4	3
4	4
5	3
4	3
4	3
4	3
5	3
3	3
5	2
3	3
2	1
4	4
4	2
5	3
4	3
3	3
5	2
4	2
5	3
5	2
4	3
4	3
4	3
5	3
4	4
3	2
5	3
5	2
4	3
3	2
4	3
5	3
5	2
5	3
5	3
4	3
5	1
5	3
5	3
3	3
4	3
5	2
5	2
5	2
5	3
5	3
4	3
5	3
5	3
3	3
5	2

5	3
5	3
3	3
4	3
5	4
5	2
3	3
5	5
5	3
5	3
4	4
5	5
4	3
3	3

@1我觉得教练A做出了正确的决定，让球员R退出	@2大多数运动员都会觉得教练A做出了正确的决定
4	4
4	4
4	4
4	4
4	4
4	4
4	4
3	3
3	3
3	3
4	4
3	3
4	4
4	4
5	5
5	5
4	4
5	5
4	4
4	4
3	3
3	2
4	3
4	4
2	3
5	4
4	4
5	5
4	4
5	4
5	5
5	5
4	3
3	3
4	4
4	4
1	1
4	4
1	3
4	4
5	5
5	5
5	5
3	3
4	4
5	5
5	5
4	4
5	5
4	4
4	4
3	2
3	3
3	3
4	5

4	4
3	3
3	3
3	3
4	4
5	5
4	4
5	5
4	3
5	4
4	3
5	5
2	3
3	3

@3我觉得运动员M应该在本赛季的第一场比赛中	@4大多数运动员会觉得运动员M应该在本赛季的
2	2
4	4
4	4
3	3
3	3
4	4
3	4
4	4
4	4
4	4
4	4
3	3
4	4
3	4
5	5
5	5
2	4
5	5
4	4
4	4
3	2
3	3
5	5
4	4
4	4
4	4
2	3
5	5
4	4
3	3
5	5
4	5
2	3
3	3
5	5
4	4
3	3
4	4
3	3
4	4
5	5
5	5
3	3
3	3
4	4
4	4
4	4
3	3
5	5
4	3
2	3
5	5
3	3
3	3
4	5

4	4
3	3
3	3
3	3
4	4
4	5
3	3
1	1
3	4
4	4
5	5
4	4
5	2
3	3

@5我觉得运动员O应该在半决赛中复出。	@6大多数运动员认为运动员O应该在半决赛中复出
2	2
4	4
4	4
3	3
3	3
4	4
4	4
4	4
4	4
4	4
4	4
3	3
4	4
3	4
5	5
5	5
4	3
5	5
4	4
4	4
3	2
3	3
5	5
4	4
4	4
4	4
3	3
5	5
4	4
3	3
5	5
5	5
4	3
3	3
5	5
4	4
3	3
4	4
3	3
3	3
5	5
5	5
3	3
3	3
2	2
4	4
4	4
3	3
5	5
3	3
3	3
4	5
3	3
3	3
5	5

2	2
3	3
3	3
3	3
4	5
4	5
3	3
1	1
3	4
3	3
5	5
4	4
3	3
3	3

@7我觉得应该由教练而不是运动员R来决定是否让	@8大多数运动员都认为，是否应该返回赛场应该
4	4
2	3
3	3
3	3
3	3
3	4
2	2
4	2
4	3
4	2
4	4
2	2
4	4
4	2
5	5
5	5
3	3
5	5
4	2
3	3
3	4
1	3
4	4
3	3
5	5
3	2
3	3
4	4
3	3
2	2
4	4
2	1
4	2
4	4
5	5
5	4
3	4
3	3
3	3
5	5
1	1
1	1
2	1
3	3
4	4
4	5
4	5
3	3
3	3
3	3
4	4
1	1
4	4
3	3
3	3

4	4
3	3
3	3
3	3
4	4
5	5
3	3
5	5
4	4
3	1
3	3
2	2
3	4
3	3

@9我觉得运动员H应该把症状告诉他的教练。	@10大多数运动员都认为运动员H应该将症状告诉
4	4
5	5
4	4
3	4
3	3
5	5
4	4
3	3
3	3
3	3
4	4
3	3
4	4
4	4
5	5
5	5
3	3
5	4
4	4
4	4
4	4
3	3
5	5
3	3
5	5
4	4
4	4
5	5
4	4
5	4
5	5
5	5
4	4
4	4
5	5
5	5
4	3
4	4
3	3
5	5
5	5
5	5
4	5
4	4
4	4
5	5
5	5
4	4
5	5
4	4
4	4
1	1
4	4
3	3
4	4

4	4
3	3
3	3
3	3
5	5
5	5
3	3
5	5
5	5
4	5
3	2
5	5
3	3
3	3

@40嗅觉异常	@40味觉异常	@40健忘症记忆缺失	@40视觉模糊	@40眼眶淤青
0	0	1	1	1
0	0	1	1	0
0	0	1	1	0
0	0	1	1	1
0	0	0	1	1
0	0	1	1	1
0	0	1	0	0
0	0	1	1	0
0	0	1	1	0
0	0	1	1	0
0	0	1	0	0
1	1	1	1	1
0	0	1	1	1
0	0	1	1	0
1	1	1	1	1
0	0	1	1	0
0	0	1	0	0
0	0	1	1	0
0	0	1	1	0
0	0	1	1	0
0	0	0	1	0
0	0	1	1	0
0	0	0	0	0
0	0	1	0	0
0	0	1	1	0
0	0	1	1	0
0	0	0	0	0
0	0	1	0	0
0	0	0	0	0
0	0	1	0	0
0	0	1	1	0
0	0	1	1	1
0	0	1	0	0
0	0	1	1	0
0	0	1	0	0
0	0	1	0	0
0	0	1	0	0
1	1	1	1	1
1	1	1	1	1
0	0	1	1	0
0	0	1	1	0
1	1	1	1	0
0	0	1	1	0
0	0	0	1	0
0	0	1	1	0
0	0	1	1	0
0	0	1	1	0
0	0	1	1	0
0	0	1	1	0
1	1	1	1	0
1	1	1	1	1
1	1	1	1	1
1	1	1	1	1
1	1	1	1	0
0	0	1	1	0

0	0	1	1	0
0	0	1	0	0
0	0	1	1	0
0	0	1	1	0
1	1	1	1	1
0	0	0	0	0
1	1	1	1	1
1	1	1	1	1
0	0	1	1	0
0	0	1	1	0
0	1	0	0	0
0	0	1	0	0
1	1	1	1	1
0	0	1	1	0

@40胸痛	@40意识混乱	@40头晕	@40头疼	@40失去知觉	@40恶心	@40鼻出血
0	1	1	1	1	1	0
0	1	1	1	0	1	0
0	1	1	1	0	1	0
0	1	1	1	0	1	0
0	1	1	1	0	1	1
0	1	1	1	0	1	0
0	0	1	1	0	1	0
0	1	1	1	0	1	0
0	1	1	1	0	0	0
0	1	1	1	0	0	0
0	1	1	1	0	1	0
0	1	1	1	1	0	0
0	1	1	1	0	1	0
0	1	1	1	1	1	1
1	1	1	1	1	1	1
0	1	1	1	0	1	0
0	0	0	0	0	0	0
0	1	1	1	0	1	1
0	1	1	1	0	1	0
0	0	1	0	0	1	0
0	1	0	1	0	1	0
0	1	1	1	0	1	0
0	1	1	1	0	1	0
0	1	1	1	0	1	0
0	0	1	1	0	1	0
0	1	1	1	0	1	0
0	0	1	0	0	0	0
0	1	1	1	0	1	0
0	0	1	1	0	1	0
0	0	1	1	0	1	0
0	1	1	1	0	1	0
0	1	1	1	0	1	0
0	0	1	1	0	1	0
0	0	1	1	0	1	0
0	0	1	1	1	1	0
0	1	1	1	0	1	0
0	0	0	0	0	0	0
1	1	1	1	1	1	1
1	1	1	1	1	1	1
0	1	1	1	0	0	0
0	1	1	1	0	1	0
0	0	1	1	0	1	0
0	1	1	1	0	0	0
0	1	1	0	0	0	0
0	1	1	1	0	1	0
0	0	1	1	0	1	0
0	1	1	1	1	0	0
0	1	1	1	1	0	0
0	0	1	1	0	1	1
0	1	1	1	1	1	0
0	0	1	1	0	0	0
1	1	1	1	1	1	1
0	1	1	1	1	1	1
0	1	1	1	1	1	0
0	1	1	1	0	1	0

0	1	1	1	0	1	0
0	1	1	1	0	0	0
0	1	1	1	0	0	0
0	1	1	1	0	1	0
0	1	1	1	0	0	0
1	1	1	1	0	1	0
1	1	1	1	1	1	1
1	1	1	1	1	1	1
0	1	1	1	0	1	0
0	1	1	1	1	0	0
0	0	0	0	0	0	0
0	0	0	1	1	0	0
1	1	1	1	1	1	1
0	0	1	1	0	1	0

@40上肢麻木刺痛	@40颈部剧烈烧灼痛	@40睡眠障碍	@40颈部活动范围受限
0	0	0	1
0	0	0	0
0	1	1	1
0	0	1	0
1	0	0	0
0	0	1	0
0	0	0	0
0	0	1	0
0	0	0	0
0	0	0	0
0	0	1	0
0	0	0	0
0	0	1	0
0	0	1	1
1	1	1	1
0	0	0	0
0	0	0	0
0	0	1	1
0	0	1	0
0	0	0	1
0	0	0	0
0	0	1	1
0	0	0	0
0	0	1	1
0	0	1	0
0	0	1	0
0	0	0	0
0	0	1	0
0	0	0	0
0	0	1	0
0	0	1	0
0	0	1	1
0	0	1	0
0	0	0	0
0	0	0	0
0	0	1	0
0	0	0	0
1	1	1	1
1	1	1	1
0	0	1	1
0	0	0	0
1	1	1	0
0	0	0	0
0	0	0	0
0	0	0	0
0	0	0	0
0	0	0	0
0	0	0	0
0	0	1	1
0	0	0	0
0	0	0	0
1	1	1	1
0	0	1	1
0	0	1	0
0	0	0	0

0	0	0	0
0	0	1	0
0	1	1	0
0	0	0	0
0	0	0	0
0	0	0	0
1	1	1	1
1	1	1	1
0	0	0	0
0	0	1	0
0	0	0	0
0	0	0	0
1	1	1	1
0	0	1	1
